# Assessing imaging performance of ultrasound systems using a random hypoechoic sphere phantom with freehand scanning

**DOI:** 10.1002/mp.70278

**Published:** 2026-01-15

**Authors:** Baihui Yu, Dufan Wu, Cristel Baiu, Zheng Feng Lu

**Affiliations:** ^1^ Department of Radiology The University of Chicago Chicago Illinois USA; ^2^ Department of Radiology The Ohio State University Columbus Ohio USA; ^3^ Department of Medical Physics University of Wisconsin Madison Wisconsin USA

**Keywords:** performance evaluation, quality control, ultrasound

## Abstract

**Background:**

Diagnostic ultrasound is rapidly evolving, and the increasing complexity of ultrasound systems underscores the importance of robust quality assurance (QA) and quality control (QC) methods. Current methods typically rely on manual image acquisition and subjective evaluation, making them operator‐dependent, poorly reproducible, and challenging for longitudinal tracking. Efforts have been made to develop automated ultrasound QA/QC methods for more quantitative and reproducible outcomes. One such approach utilized the Random Hypoechoic Sphere Phantom (RHSP) to evaluate the detectability of ultrasound systems by measuring the human observer‐related Lesion Signal‐to‐Noise Ratio (LSNR). A practical manual scanning method was developed for the RHSP, which eliminated the need for a mechanical guide to control transducer translation. However, this approach requires maintaining a uniform translation speed and a small translation distance between successive images. Broader adoption of the RHSP also requires easily accessible automated analysis software.

**Purpose:**

To improve the practicality of using the RHSP for ultrasound performance evaluation in clinical settings, we developed an automated analysis method tailored for freehand scanning. We validated the method by evaluating ultrasound system performance under various acquisition settings, identifying sources of variability, and analyzing their impact on LSNR measurements.

**Methods:**

The RHSP consisted of 2 mm diameter spheres at 20% volume fraction. It was scanned by moving the transducer by freehand during a cine‐loop acquisition. Volumetric images were generated by stacking the elevational frames. After denoising and homogeneity correction, the spheres were segmented in 3D using depth‐adaptive thresholds from a Gaussian‐Mixture Model. LSNR was computed for each segmented sphere, and LSNRs at similar depth were averaged to generate an LSNR vs. depth curve.

The algorithm was validated by computing the LSNR vs. depth and sphere‐count vs. depth curves for various acquisition settings, with the following factors changing one at a time: nominal frequency, imaging modes and compound techniques, transmit power, gain, dynamic range, and transmit focal depth. Variability of the algorithm was assessed using repeated scans performed by different operators and at various transducer translation speeds.

**Results:**

All LSNR results closely matched visual assessments and followed expectations when changing acquisition settings. LSNR values improved with higher nominal frequency, harmonic imaging with compound, higher transmit power, and decreased dynamic range; gain had minimal effect. The best LSNR also aligned with the transmit focal depths set by the operator. The variabilities of LSNR were from 2.5% to 6.9% intra‐operator, 5.2% inter‐operator, and 5.5% due to transducer translation speed. These variabilities of the LSNR were much smaller than those of the corresponding sphere counts, demonstrating the proposed algorithms’ robustness against potential variations produced by freehand scanning.

**Conclusions:**

The automated analysis method for the RHSP with freehand scanning provides an accurate and stable quantitative assessment of lesion detectability for evaluating ultrasound performance, and is feasible in clinical settings. It demonstrated robustness by achieving approximately 5% variability in LSNR across various transducer translation speeds and different operators. With such robustness, this approach simplifies ultrasound performance evaluation and holds promise for integration into routine ultrasound QA/QC in a clinical environment.

## 1 INTRODUCTION

Quality assurance/quality control (QA/QC) is important to ensure consistent imaging performance of diagnostic ultrasound systems.^1^, ^2^, ^3^ For gray‐scale ultrasound, various phantoms have been designed for specific characteristics assessment.^4^, ^5^ For example, the Multi‐Purpose Multi‐Tissue Ultrasound Phantom, with various built‐in targets, has been widely used to evaluate a series of image quality metrics in routine QC.^6^ However, one limitation of such phantoms is that the targets are cylindrical in shape and cannot assess elevational spatial resolution. The Edinburgh Pipe Phantom consists of diagonally positioned pipes of various sizes, which can assess spatial resolution in all three directions, including the elevational direction.^7^, ^8^ However, the measurement procedure is time‐consuming, and the current analysis method is subjective and operator‐dependent. A cost‐effective phantom is needed—one that enables easy and rapid scanning for image acquisition and automated analysis to effectively assess the detectability of an ultrasound system in clinical settings.

A sphere phantom was developed to assess the ability of an ultrasound system to delineate tumor‐like objects from surrounding soft tissue. The detectability of each sphere is expressed as the Lesion Signal‐to‐Noise Ratio (LSNR), which correlates directly with human observer‐related detectability.^9^, ^10^ An earlier version of the sphere phantom contained only a single layer of co‐planar, uniformly distributed spheres.^11^ It was difficult to manufacture, and it required adjusting the transducer's orientation by hand to perfectly align the scan plane with the sphere plane. Subsequently, a randomly distributed sphere phantom was proposed, along with a mechanical device to translate the transducer.^12^ This device controlled the scanning increment within ¼ of the sphere diameter to ensure that LSNRs were derived from the scanning plane passing through the centroid of each sphere. The mechanical translation method increased the total cost of the phantom system and increased the scanning time to acquire images, leading to limited usage of the phantom in routine QC in a clinical environment.^12^, ^13^, ^14^ To address these challenges, the cine loop recording method with manual transducer translation was proposed.^13^, ^15^ However, this approach still required maintaining a uniform translation speed and interframe distances within ¼ of the sphere diameter.^13^, ^15^ In addition, the initial version of Random Hypoechoic Sphere Phantoms (RSHPs) contained sparsely arranged spheres, leading to increased uncertainty of LSNR calculation.^12^, ^13^ A denser phantom is expected to improve the accuracy of LSNR analysis.

In this study, we utilized a RHSP containing 2 mm‐diameter spheres at a volume fraction of 20%, which is significantly denser than the volume fractions used in earlier‐published RHSPs.^12^, ^13^ Volume fraction is defined as the total volume of all hypoechoic spheres divided by the total phantom volume.^12^ The phantom was scanned by moving the transducer manually while recording images in a cine acquisition. The transducer translation speed can vary, and it doesn't need to follow the requirement of interframe distances within ¼ of the sphere diameter. A novel depth‐adaptive threshold‐based 3D segmentation method was developed to segment each sphere from the background. Then the LSNR for each sphere was calculated within the plane where the maximum sphere cross‐section was located. Average LSNR values were calculated for spheres in the same depth slab to derive the LSNR vs. depth curve. We evaluated the LSNR for acquisitions under different settings, including frequency, transmit power, and overall gain. The reproducibility of the method was evaluated among inter‐ and intra‐operator acquisitions, as well as acquisitions with various transducer translation speeds.

Our main contributions are summarized as follows: (1) an improved manual translation method for the RHSP that no longer requires a steady transducer translation speed; (2) a novel method to robustly segment spheres for LSNR calculation; (3) the first study to evaluate both intra‐operator and inter‐operator variability in LSNR measurements using the RHSP.

## 2 METHODS

### 2.1 Phantom description

**FIGURE 1:**
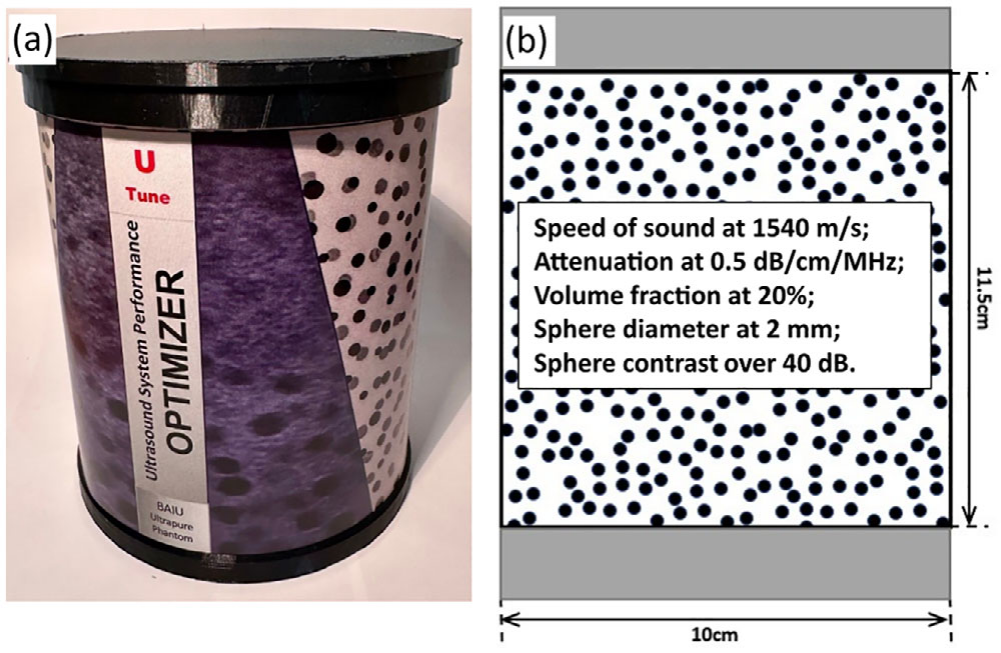
(a) Photograph of the RHSP with 2 mm‐diameter spheres; (b) Diagram of the RHSP with 2 mm‐diameter spheres.

A prototype RHSP, manufactured by UTune LLC was utilized in this study. The phantom contains randomly distributed, high‐contrast spheres with a diameter of 2 mm for adequate performance assessment of the high‐frequency range between 7 MHz and 18 MHz.^13^ A picture and schematic diagram of the RHSP are shown in Figure 1. The background medium consists of water‐based tissue‐mimicking material with a sound propagation speed of 1540 m s^−1^ and an attenuation coefficient slope of 0.5 dB cm^−1^ MHz^−1^. The spheres have a backscatter coefficient of −40 dB relative to the background material. The number of spheres per milliliter is around 48, and the volume fraction of spheres is around 20%. The phantom is designed to be simple to produce and cost‐effective.

### 2.2 Image acquisition

Instead of acquiring images in conjunction with a mechanical guide, we acquired cine loop images through freehand scanning by manually translating the transducer. During the translation, the transducer was kept perpendicular to the phantom's surface, and moved slowly and steadily in a direction normal to the image plane. Notably, our method does not require following the recommendation in IEC TS 62791:2022^13^ to keep the imaging interval between adjacent frames within 1/4 of the sphere diameter. In our experiments, changing the transducer translation speed from approximately 0.5 cm/s to 1.5 cm/s when the frame rate was 15 images/s (imaging interval from 0.3 mm to 1 mm) did not impact the LSNR results. Moreover, our proposed analysis method is robust to variations in uneven transducer translation speed typically observed in freehand scanning.

### 2.3 Analysis method

#### 2.3.1 Overview

**FIGURE 2:**
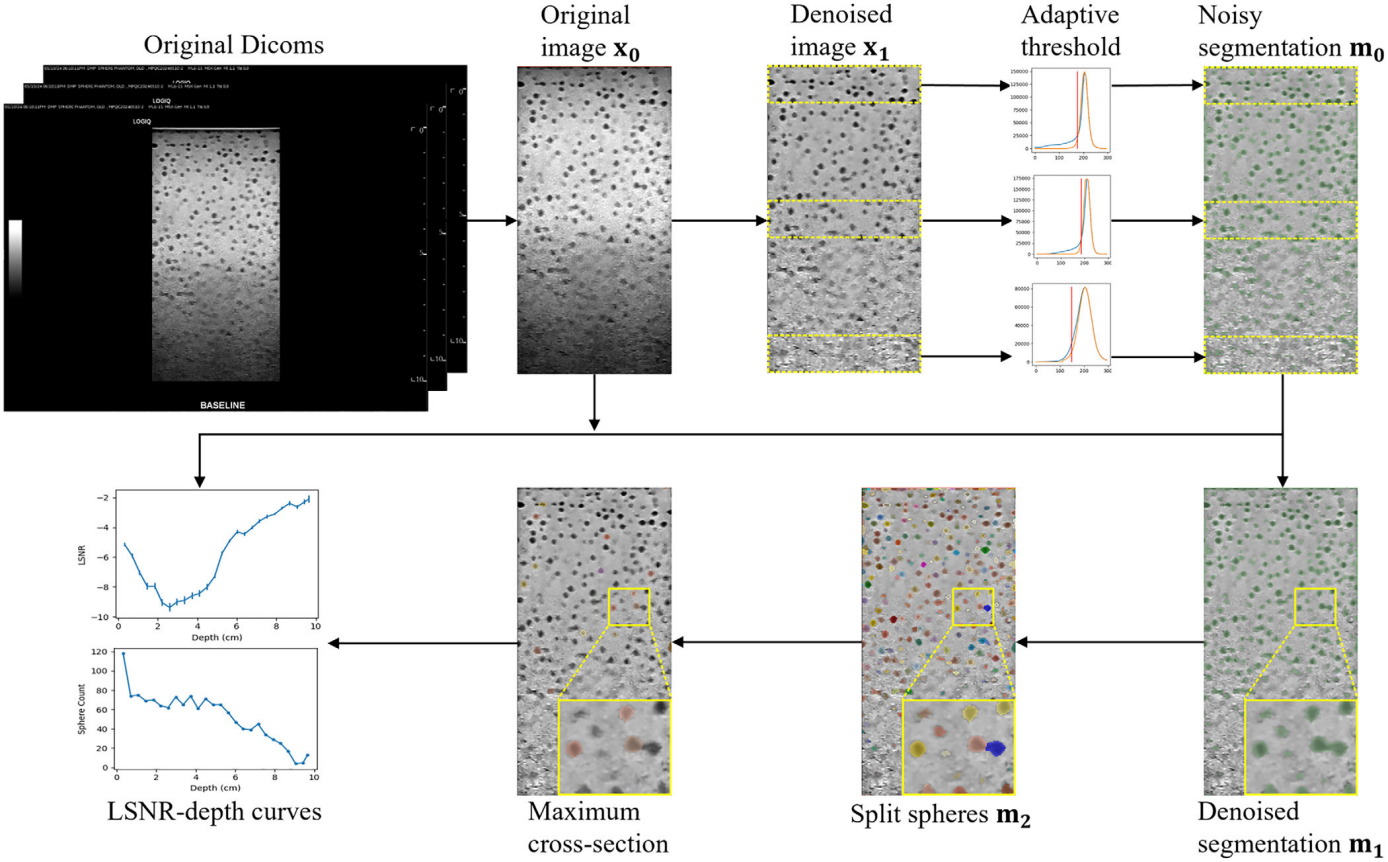
Algorithm flowchart. The final LSNR vs. depth curve is calculated on the original image $x_{0}$ with sphere mask derived from the maximum cross‐section and background mask derived from $m_{0}$.

The purpose of the image analysis is to calculate the LSNR vs. depth curve from the acquired image stack automatically. Following Madsen et al.,^12^ we first segment all the detected spherical voids and then calculate the LSNR for each sphere against its surrounding background. A flowchart is demonstrated in Figure 2. First, the effective volume is extracted from the acquired ultrasound images. Second, the volume is preprocessed by 3D non‐local means (NLM) denoising^16^ and N4 bias field correction^17^ for noise and heterogeneity reduction. Third, an improved adaptive thresholding algorithm is proposed to find the best threshold at each depth to segment the spherical voids from the background. Fourth, any connected spheres are further split by the watershed algorithm.^18^ Fifth, the maximum cross‐section of each sphere is identified along the elevational direction. Last, the LSNR of each sphere is calculated at its maximum cross‐section, and the LSNR vs. depth curve is depicted by averaging the LSNRs at the same depth slab.

**FIGURE 3:**
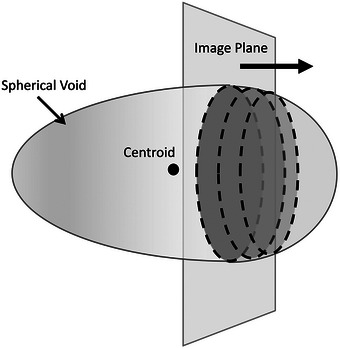
The asymmetry of the acquired sphere image resulted from freehand scanning. The mass centroid of the sphere lay outside the image plane where the maximum cross‐section of the sphere is located.

Although the proposed analysis method uses the same LSNR vs. depth framework as in Madsen et al.,^12^ it differs substantially in the following aspects:

First, unlike mechanical scanning where the elevational separation between images is kept the same, freehand scanning is used in our method where the transducer translation speed is not uniform. As demonstrated in Figure 3, freehand scanning can cause the reconstructed sphere to appear asymmetrical along the transducer translation direction. Consequently, its mass centroid may fall outside the image plane that intersects the true center of the sphere, leading to the diameter of the mass centroid sphere being smaller than the actual diameter. Hence, using the centroid for LSNR calculation as suggested in Madsen et al.^12^ may lead to smaller cross‐sections being used as the spherical voids. To alleviate this bias, we propose to find the cross‐section with the maximum area along the elevational direction, which best represents the desired diameter of the spherical void. Compared to the mass centroid‐based approach, our method of finding the maximum cross‐section requires more precise segmentation of each spherical void, because it compares the segmentation in each slice and identifies the one slice with the maximum area, rather than averaging them across all slices to determine the mass centroid.

Second, the phantom used in this study has a much higher volume fraction of spherical voids compared to the one used in Madsen et al.^12^ While a higher sphere density leads to a more stable estimation of the LSNR, it introduces challenges for image analysis. One such challenge is the adaptive thresholding to segment the spheres from the background. The method proposed in Madsen et al.^12^ estimated the threshold by taking the mean and standard deviation (STD) of all pixels at each depth interval, under the assumption that they are a good approximation of the background distribution. However, with the higher volume fraction of spherical voids in our phantom, the mean and STD of all the pixels are significantly impacted by the spherical voids and no longer represent the background. Hence, an improved adaptive thresholding method was proposed using a Gaussian‐Mixture Model.

Another major challenge is that adjacent spheres may appear connected after segmentation due to the high sphere density. To resolve this issue, a watershed algorithm was employed to separate connected spheres, and thresholds based on the area of the maximum cross‐section were used to exclude spheres that remained connected or became fragmented due to failed segmentation by the watershed algorithm. Details of the watershed algorithm are given in section 2.3.5.

#### 2.3.2 Volume extraction

The frames acquired from a cine loop are stacked into a volumetric image for analysis. The original DICOM images have an overlay around the field of view (FOV), which needs to be removed first. The first few rows of the FOVs are susceptible to surface reflections of the ultrasound and should be excluded. Similarly, the last few rows are also susceptible to poor signals and are excluded from the analysis as well. To extract the effective volume, we search for the first row/column that consists of all zeros for at least 50 pixels from the image center in each of the four directions. The first and last 10 rows in the depth direction are excluded from image analysis. This extracted volume before any preprocessing is denoted as $x_{0}$, as shown in Figure 2 and is used for LSNR calculations.

#### 2.3.3 Preprocessing

Three‐dimensional (3D) NLM denoising, with an isotropic searching window size of 9 pixels and a comparing patch size of 3 pixels is used to reduce image noise. The denoising strength σ is taken as $\sigma =\sigma_{0}/8$, where $\sigma_{0}$ is the STD of the entire image $x_{0}$. N4 bias field correction, which was initially designed for heterogeneity correction in magnetic resonance imaging (MRI) is employed following NLM to improve the homogeneity of the image. Because ultrasound images exhibit much higher heterogeneity along the depth direction compared to the lateral and elevational directions, we used 8 control points along the depth and 4 control points along each of the other two directions. The image after the preprocessing steps is denoted as $x_{1}$ as shown in Figure 2, and is used for spherical void segmentations.

Non‐local means perform image denoising by averaging pixels that have similar surrounding patches, thus it can achieve better noise reduction and edge preservation compared to classic local filters such as Gaussian blur. In practice, it averages each pixel with all its surrounding pixels within a certain distance, where the averaging weights are determined by the similarities between the patches around the central pixel and each surrounding pixel. It encourages more aggressive averaging between patches with the same underlying structure, while discouraging averaging between different patches. Thus, structures such as edges are better preserved.

#### 2.3.4 Initial segmentation

Despite N4 correction, there is still substantial inhomogeneity along the depth direction due to signal attenuation. Following Madsen et al.,^12^ we divided the image into 10 slabs along the depth direction and calculated an adaptive threshold for each slab to segment the spherical void from the background. As discussed in 2.3.1, due to the large fraction of spherical voids in our phantom, the distribution of all the pixel values within a slab no longer represents the distribution of the background, and thus cannot be used to derive the adaptive threshold.

To tackle this challenge, we modeled the pixel value distribution of each slab as a mixture of two Gaussian distributions, where the background and the spheres are each considered as a Gaussian distribution. Direct mixture Gaussian fitting may be susceptible to instability because the number of pixels from the spheres is substantially less than the background. Instead, a simpler yet robust method was proposed to find the distribution of the background to determine the threshold. Given the fact that signals from the background are dominant and the background has larger pixel values than the spheres, we assumed that the peak of the all‐pixel histogram is from the background distribution. Furthermore, it is also assumed that all the pixels larger than the histogram peak are from the background.

**FIGURE 4:**
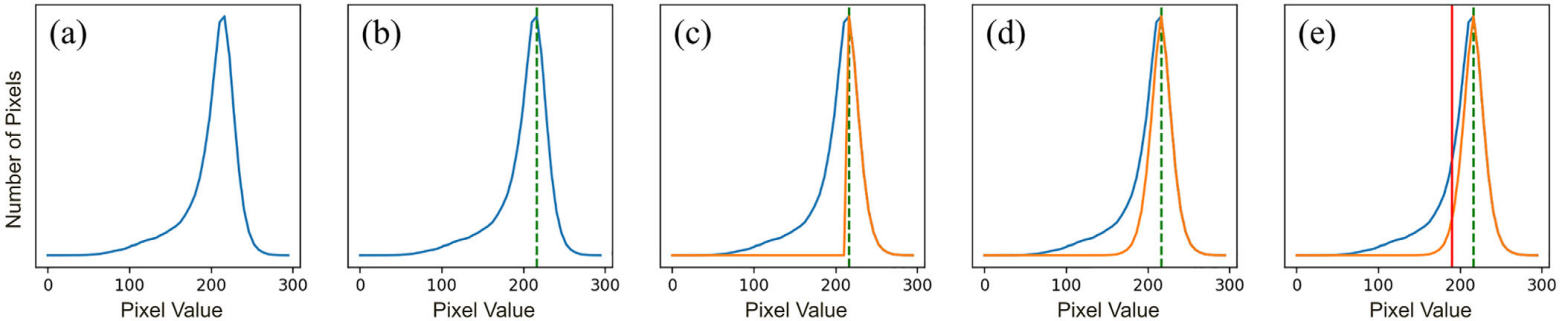
Adaptive threshold for slab k. (a) pixel value histogram $P_{k}$; (b) peak value $\mu_{k}$, marked by the dashed green line; (c) right half of the background distribution; (d) full background distribution $N(\mu_{k},\sigma_{k}^{2})$ by mirroring around $\mu_{k}$; (e) the threshold $\mu_{k}-2\sigma_{k}$, marked by the solid red line.

As shown in Figure 4, the adaptive threshold for each slab k is estimated by the following steps.

First, given the pixel value histogram $P_{k}$, find its peak value

(1) $$\mu_{k}=\arg\max_{x}P_{k}\left(x\right)$$

where $\mu_{k}$ will be considered as the mean of the background Gaussian distribution $N(\mu_{k};\sigma_{k}^{2})$.

Second, all the pixel values larger than $\mu_{k}$ are assumed to be from the background, and this set of pixels $S_{k}=\left\{x|x\geq \mu_{k},x∼P_{k}\right\}$ constructs the right half of the background distribution $N(\mu_{k};\sigma_{k}^{2})$.

Third, to calculate $\sigma_{k}^{2}$, although it is possible to calculate from the set $S_{k}$, it is more straightforward to mirror every pixel in $S_{k}$ around $\mu_{k}$ to get the full distribution, i.e.:

(2) $$S_{k}^{\prime }=\left\{x|x\geq \mu_{k},x∼P_{k}\right\}\cup \left\{2\mu_{k}-x|x\geq \mu_{k},x∼P_{k}\right\}$$

which means that for every value x in the set $S_{k}$, we add a mirrored value $2\mu_{k}-x$ to the set.

Finally, the STD of the background distribution can be calculated by taking the STD of all the elements in the set $S_{k}^{\prime }$, i.e. $\sigma_{k}=STD\left(\left\{x|x\in S_{k}^{\prime }\right\}\right)$. And the threshold for the slab k is chosen empirically as:

(3) $$T_{k}=\mu_{k}-2\sigma_{k}$$

Any pixels below the threshold $T_{k}$ are considered as the spheres, and the segmentation map is denoted as $m_{0}$, which is used to mask the background in LSNR calculations. Three iterations of binary opening are applied to $m_{0}$ to remove noise in the segmentation mask, and the result is denoted as $m_{1}$. $m_{1}$ is subsequently used in the following morphological operations to segment each individual sphere.

#### 2.3.5 Split connected spheres

Due to the high‐volume fraction of spheres, many segmented spheres are likely to be connected in the initial segmentation $m_{1}$. Because we need to measure LSNR at the maximum cross‐section of each sphere, it is necessary to separate the connected spheres. We employed the following distance transformation and watershed algorithm to achieve this goal.

First, a distance transformation is done for all the sphere pixels in $m_{1}$. In the transformation, the Euclidean distance from each sphere pixel to its nearest background pixel is calculated. Second, pixels with the local maximum distances are marked as the seed points for the watershed algorithm. The minimum distance between two seed points is set to 5 pixels empirically. Last, the watershed algorithm is executed with the proposed seeds, and the regions originating from different seeds are marked as different spheres. This multi‐label segmentation, splitting connected spheres is denoted as $m_{2}$.

The watershed algorithm is a classic image processing method used to segment connected regions into distinct regions. It treats connected spheres as water basins and simulates the gradual filling of water into each basin until water from different basins meet each other. Practically, the watershed algorithm is done by: 1) taking the distance transform of the mask; 2) finding seed points using local maximum distance; 3) building a priority queue based on the distance; 4) iteratively fetching pixels with the largest distance in the queue and assigning them the labels from neighboring pixels, which is repeated until only pixels with conflicting neighbors are left. The labeled pixels form the new segmentation, while connected spheres are split.

#### 2.3.6 Identify the maximum cross‐section along elevation

For each segmented sphere in $m_{2}$, its cross‐section with the largest area along the elevational direction is identified. Because the watershed algorithm does not guarantee the splitting of all connected spheres, and it may also over‐split a single sphere into smaller fragments, an area‐based constraint is further applied to filter out both the connected and fragmented sphere segmentations.

Specifically, given the expected sphere diameter d in the phantom, denoting the area of the maximum cross‐section for a sphere i as $s_{i}$, we only keep the spheres that satisfy:

(4) $$\frac{\alpha_{1}\pi d^{2}}{4}\leq s_{i}\leq \frac{\alpha_{2}\pi d^{2}}{4},$$

where $\alpha_{1}=0.5$ and $\alpha_{2}=1.25$ in our studies. $\pi d^{2}/4$ is the expected area of the maximum cross‐section of the spheres.

#### 2.3.7 Calculate the LSNR

For each eligible sphere i according to Equation (4), its center $\left(x_{i},y_{i},z_{i}\right)$ is first calculated. The elevation coordinate $z_{i}$ is taken as the slice where the maximum cross‐section is. The $x_{i}$ and $y_{i}$ are the averaged coordinates of all the pixels that belong to the sphere i in the maximum cross‐section. Then the LSNR for the sphere i is calculated as:

(5) $$LSNR_{i}=\frac{S_{\mathrm{fi}}-S_{\mathrm{bi}}}{\sigma_{\mathrm{bi}}},$$

where $S_{\mathrm{fi}}$ is the signal of the sphere, $S_{\mathrm{bi}}$ is the signal of the background, and $\sigma_{\mathrm{bi}}$ is the noise of the background. All the values are calculated from the original cropped image $x_{0}$ as follows:

Sphere signal $S_{\mathrm{fi}}$: sphere pixels are found from the processed segmentation map $m_{2}$. For sphere i, we first take its segmentation mask within the maximum cross‐section slice $z_{i}$. Then this mask is eroded by 3 pixels within the slice, and all the pixels in the slice $z_{i}$ inside the eroded mask are considered sphere pixels. $S_{\mathrm{fi}}$ is calculated by taking the mean of all the pixels for sphere i.

Background signal $S_{\mathrm{bi}}$ and STD $\sigma_{\mathrm{bi}}$: a cylindrical mask $m_{i}$ with a diameter of 4d (d is the designed diameter of the spheres) and centered at $\left(x_{i},y_{i}\right)$ is created along the elevational direction across all the slices. The cylindrical mask across all frames was used as the background because 1) the background of the phantom is uniform and the signal at the same distance to the transducer should be similar across all frames; 2) it will greatly increase the number of samples for background and reduce the uncertainty in estimating the background mean and noise. All the pixels within the cylinder $m_{i}$ but outside the initial noisy mask $m_{0}$ dilated by 1 pixel are considered as the surrounding background for the sphere i. The one‐pixel dilation is to exclude as many sphere pixels as possible. Then $S_{bi}$ and $\sigma_{bi}$ are calculated from these pixels. Here, the noisy mask $m_{0}$ instead of the denoised $m_{1}$ is used to exclude sphere pixels as much as possible.

After the $LSNR_{i}$ is calculated for each eligible sphere, the whole LSNR vs. depth curve is depicted by dividing the image into 25 uniform slabs along the depth direction. For the 10 cm depth image, each slab has around 0.4 cm extent in the axial direction. Assuming the kth slab starts from $y_{k}$ and ends at $y_{k+1}$, then the LSNR is calculated as:

(6) $$LSNR_{k}=LSNR\left(\frac{y_{k}+y_{k+1}}{2}\right)=\frac{1}{N_{k}}\;\sum_{y_{k}\leq y_{i}<y_{k+1}}LSNR_{i}$$

where $N_{k}$ is the number of spheres detected in the kth slab. $y_{i}$ is the center of the ith sphere along the depth direction. $LSNR_{i}$ is the LSNR of the ith sphere given in Equation (5).

The uncertainty of the $LSNR_{k}$ is calculated as:

(7) $$\varepsilon \left(\frac{y_{k}+y_{k+1}}{2}\right)=\frac{\sigma_{LSNR_{k}}}{\sqrt{N_{k}}},$$

where $\sigma_{LSNR_{k}}$ is the standard deviation of the LSNRs from all the spheres in the kth slab.

### 2.4 Experiments

#### 2.4.1 Accuracy validation in different configurations

The proposed method was first applied to the data acquired under various acquisition settings. To validate its accuracy, we compared the calculated LSNR curves with the visual impressions of the corresponding images. Furthermore, the differences in the LSNR curves across different acquisition settings were also evaluated against the theoretical expectations.

The phantom was first scanned on a GE Logiq E10 system by an ML6‐15 linear array transducer using cine acquisition. LSNR and sphere counts as functions of depth were evaluated by changing the following factors one at a time: nominal frequency, imaging modes and compound techniques, transmit power, gain, and dynamic range. In these experiments, the E10 system used a synthetic transmit focus, which was automatically applied by the scanner and could not be adjusted by the operator. The phantom was then scanned on a GE Logiq E9 system under different focal zone settings. Each setting had two transmit foci, which were located at 1 and 2 cm, 2 and 3 cm, and 3 and 4 cm, respectively. The experimental setup parameters are given in Table 1. The cine loop duration in the above experiments was approximately 3 seconds. The cine frame rate was automatically changed when switching from B‐mode to compounding mode, and showed minor variations across different focal zone settings. The acquisition parameters are summarized in Tables 1 and 2.

**TABLE 1:** Experiments setup parameters.

Experiments	Parameters^*^			Equipment	Transducer
Reproducibility validation	15 MHz, Harmonic with compounding, Power 100%, Gain 56, Dynamic range 63 dB			GE Logiq E10^**^	ML6‐15
Nominal frequency (MHz)	15	12	10		
Imaging modes and compound techniques	Harmonic with compounding	Harmonic without compounding	B‐mode without compounding		
Transmit power	100%	50%	24%		
Gain	56	50	44		
Dynamic range (dB)	84	63	42		
Transmit focus (cm)	1 and 2	2 and 3	3 and 4	GE Logiq E9	

* The protocol for all the acquisition settings is MSK GEN.

** The transmit focus was automatically optimized for the GE Logiq E10 system, where the transmit focus was uniformly applied across the entire depth range.

**TABLE 2:** Imaging matrix and cine settings.

Experiments	Depth (cm)	Matrix size for phantom region	Pixel size (mm^2^)	Cine frame rate (fps)^*^	Cine loop duration (sec)^*^	Number of frames
Reproducibility validation	10	358 × 711	0.14 × 0.14	15	4.13	62
Nominal frequency (MHz)				14 for all except 21 for B‐mode without compounding	3	42 for all except 63 for B‐mode without compounding
Imaging modes and compound techniques						
Transmit power						
Gain						
Dynamic range						
Transmit focus 1 and 2 cm	5	784 × 783	0.064 × 0.064	25	3.12	78
Transmit focus 2 and 3 cm				23	3.13	72
Transmit focus 3 and 4 cm				21	3.24	68

* The Cine frame rate and Cine loop duration were automatically changed for different focal zone settings.

#### 2.4.2 Reproducibility validation

We evaluated three aspects of the proposed method, including intra‐operator variability, which measures the variability among repeated scans by a single operator; inter‐operator variability, which measures the variability among different operators; and variability against different transducer translation speeds.

**FIGURE 5:**
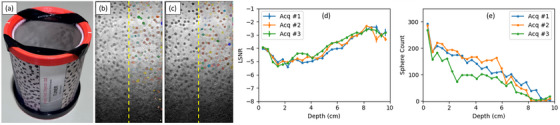
(a) phantom with tape marking the transducer coverage; (b) one frame from 6 cm coverage and the segmentation; (c) one frame from 4 cm coverage and the segmentation; (d) LSNR curves from 3 repeated scans with 6 cm coverage by Operator #1; (e) sphere counts curves from 3 repeated scans.

The phantom was scanned using a GE Logiq E10 system with an ML6‐15 transducer at 15 MHz. All cine loops had a fixed 4.13s duration and contained 62 frames. The starting and ending positions of each scan were marked by tape on the surface of the phantom, as shown in Figure 5a. By adjusting the tape positions, various scanning coverage distances were achieved within the same cine loop duration, which led to different transducer translation speeds. A single operator scanned 2, 3, 4, 5, and 6 cm coverage at increasing speeds. Two representative frames from the 4 cm and 6 cm coverage cine loops, along with their segmentations, are shown in Figures 5b and c to illustrate the differences in appearance resulting from varying transducer translation speeds. Then five operators conducted acquisitions for the 6 cm coverage using the same parameters. Each operator performed 10 repeated acquisitions to assess repeatability. Figures 5d and e present the LSNR and sphere counts as functions of depth from three repeated acquisitions of the 6 cm coverage by Operator #1.

The variability of LSNR within a single depth slab d was defined as the relative standard deviation of the LSNRs from different acquisitions:

$$RSD_{\mathrm{LSNR}}\left(d\right)=SD_{\mathrm{LSNR}}\left(d\right)/Mean_{\mathrm{LSNR}}\left(d\right)$$

The overall variability of LSNR was defined as $RSD_{\mathrm{LSNR}}\left(d\right)$ averaged over all depths d. The variability of sphere counts was defined similarly.

## 3 RESULTS

### 3.1 Accuracy validation in different configurations

#### 3.1.1 Changing transducer frequency

**FIGURE 6:**
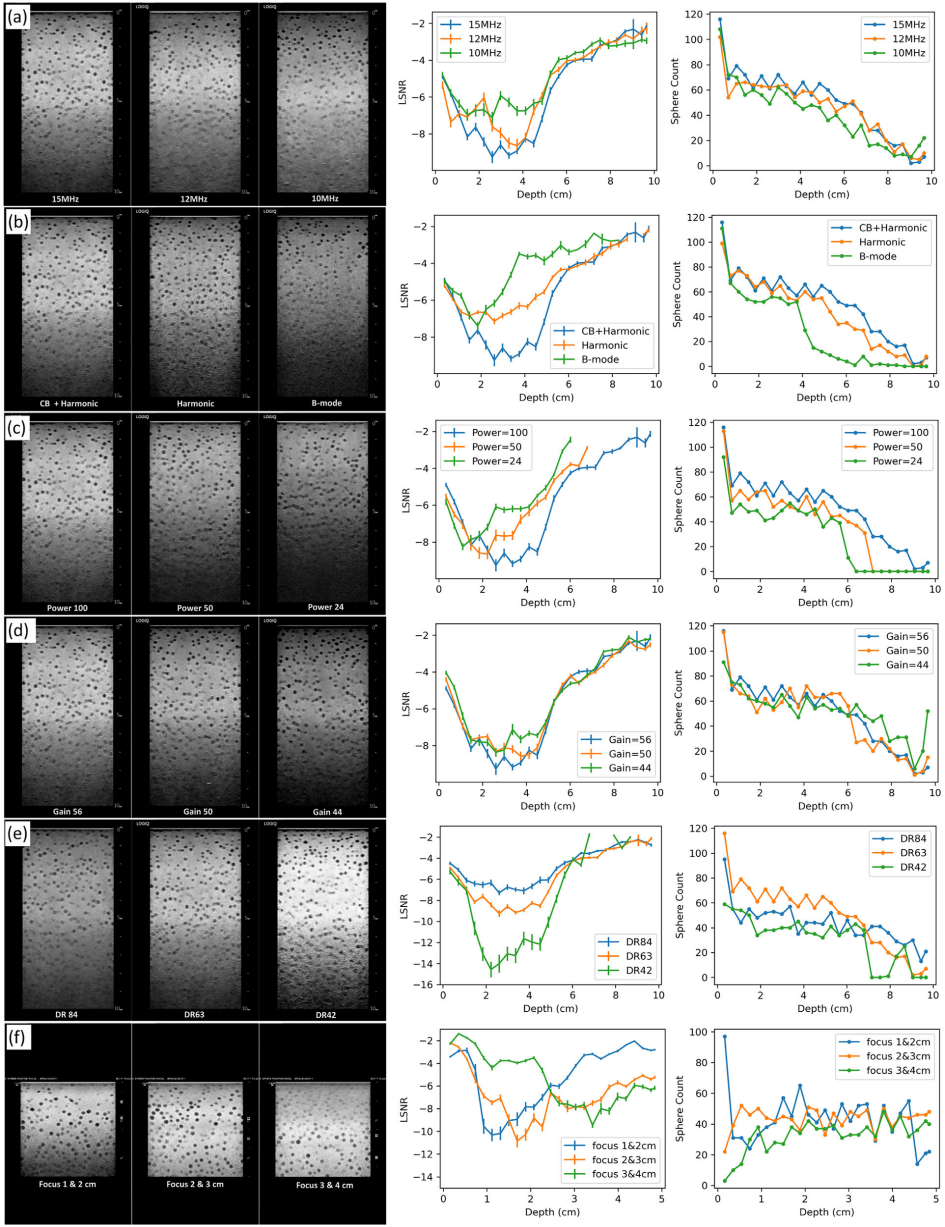
(a) changing nominal frequencies; (b) changing imaging mode and cross‐beam (CB) compound techniques; (c) changing power; (d) changing gain; (e) changing dynamic range; (f) changing dual‐focus depth. From left to right, the columns are elevational frames under different acquisitions/ settings, LSNR vs. depth curves, and sphere count vs. depth curves.

The results from different nominal frequencies of 15, 12, and 10 MHz are shown in Figure 6a. The images demonstrate better penetration at lower frequencies, with brighter signals in the deeper regions. However, at nearly all depths, the 15 MHz LSNR curve outperforms the 12 MHz curve, and the 12 MHz curve outperforms the 10 MHz curve. This is because, despite the better penetration, lower frequencies reduce spatial resolution, leading to decreased overall LSNR performance in the phantom.

#### 3.1.2 Changing imaging mode and compound techniques

The results from different imaging modes and compound methods are shown in Figure 6b (Harmonic with compounding, harmonic without compounding, and B‐mode without compounding). As expected, both visual impressions from the images and the LSNR curves agree that harmonic with compounding mode performs better than harmonic without compounding mode, and harmonic without compounding mode performs better than B‐mode without compounding mode. The improvement of the image quality is most prominent in the mid‐range (2 – 6 cm), as suggested by both visual inspection and the LSNR curves.

#### 3.1.3 Changing transducer power

The influence of different transmit powers of 100%, 50%, and 24% is given in Figure 6c. All three LSNR curves share similar values in the superficial region (< 1 cm), whereas higher transmit power leads to better LSNR at deeper depths. From the images, the spheres below 7 cm and 6 cm are hardly visible for the 50% and 24% power settings, respectively. This can be easily characterized by the sphere count curves, where the number of detected spheres is 0 when the depth exceeds 7.15 cm and 6.40 cm, respectively. The corresponding LSNR values are also absent below the specific depths because no spheres are detected.

#### 3.1.4 Changing gain

Three gain factors of 56, 50, and 44 were tested, and the results are given in Figure 6d. Despite the significantly darker image at a lower gain setting, the LSNR remains approximately the same across all three gain settings. Because gain is a uniform amplification of the echo signals at all depths, both the numerator and the denominator of the LSNR change proportionally, leaving LSNR unchanged. The LSNR curves follow this prediction, although it may be counterintuitive against the visual impression from the images.

#### 3.1.5 Changing dynamic range

Meanwhile, another parameter, dynamic range, demonstrates a different behavior compared to the gain factor. We tested three dynamic ranges of 84 dB, 63 dB, and 42 dB, and found that a smaller dynamic range led to a better LSNR, as shown in Figure 6e. This is likely due to image over‐saturation and noise value clipping, which could lead to underestimated background noise $\sigma_{\mathrm{bk}}$ in the LSNR calculation and cause a pseudo improvement of the LSNR.

#### 3.1.6 Changing transmit focus

Last but not least, a dual‐focus setting on a GE Logiq E9 system with the same ML6‐15 linear array transducer was used to evaluate the impact of the transmit focal depth setting. The depths of the transmit focus for the dual‐focus settings were 1 and 2 cm, 2 and 3 cm, and 3 and 4 cm for three repeated scans, respectively. The results are given in Figure 6f. Peak LSNRs of −10.32, −10.85, and −8.2 were achieved at 1.1 cm, 1.7 cm, and 3.8 cm, respectively, for the three scans, which complied with the image and settings. It can be observed that the 2 and 3 cm dual‐focus setting achieved the best overall LSNR within the evaluated depth range from 0 to 5 cm. It achieved only slightly worse LSNR at depths deeper than 3 cm compared to the 3 and 4 cm dual‐focus setting. In contrast, the 1 and 2 cm dual‐focus setting performed much worse at depths deeper than 3 cm. These results suggest that the 2 and 3 cm dual‐focus setting should be selected as the default preset for examinations from 0 to 5 cm based on its superior overall lesion detectability.

### 3.2 Variability source evaluation

#### 3.2.1 Intra‐operator variability

Tables 3 and 4 present the calculated intra‐operator, test‐retest variabilities, where the variabilities of the LSNRs and sphere counts were calculated among repeated acquisitions with the same transducer scanning coverage and by the same operator. In addition to the overall variability, a single point variability at the depth of 2.2 cm was also given, as this specific depth corresponded approximately to the best LSNR along the depth across all acquisitions. The overall variability of LSNR remained around 5% for most settings, while that of the sphere counts was much greater, ranging from 13% to 25%. Both LSNR and sphere counts had smaller variabilities at the 2.2 cm depth in comparison to those of other depths, but the sphere counts still showed 2 ‐ 3 times more variability than LSNR for most settings. Figures 5d and 5e also demonstrated the significantly smaller variability of LSNR compared to sphere counts.

**TABLE 3:** The intra‐operator variability for different transducer translation speeds.

	Overall variability		Variability at 2.2 cm	
Transducer coverage	LSNR	#Spheres	LSNR	#Spheres
2 cm	4.5%	22.6%	5.3%	18.3%
3 cm	3.7%	13.2%	3.6%	6.7%
4 cm	6.9%	25.5%	5.4%	10.4%
5 cm	3.1%	15.1%	4.0%	14.7%
6 cm	5.3%	17.7%	4.4%	14.2%

**TABLE 4:** The intra‐operator variability for different operators.

	Overall Variability		Variability at 2.2 cm	
Operator	LSNR	#Spheres	LSNR	#Spheres
#1	5.3%	17.7%	4.4%	14.2%
#2	3.8%	16.8%	5.6%	17.6%
#3	5.8%	17.7%	5.6%	13.3%
#4	14.2%	21.5%	9.6%	13.6%
#5	2.5%	9.1%	2.1%	5.8%

#### 3.2.2 Transducer translation speed‐induced variability

**FIGURE 7:**
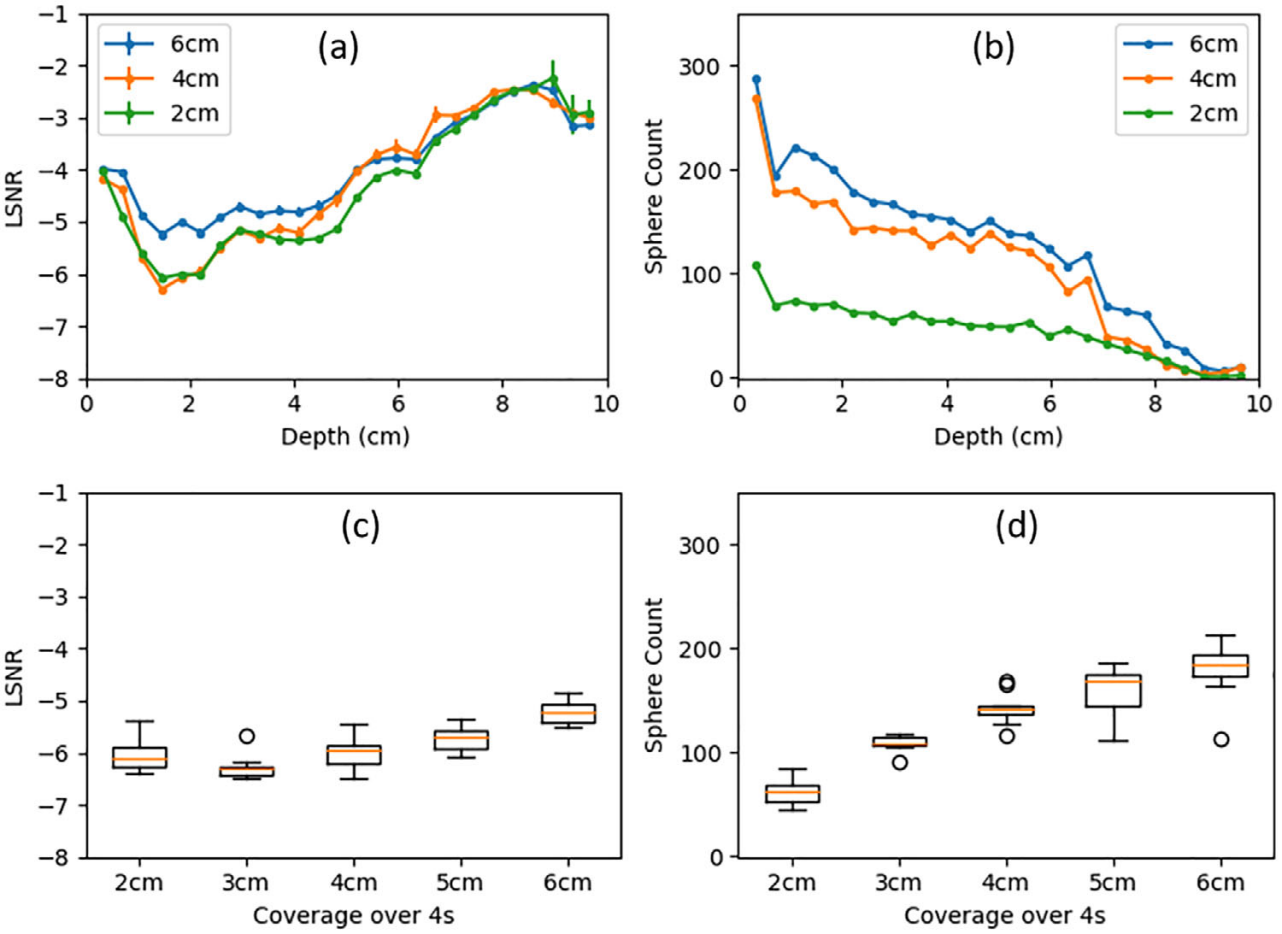
Transducer translation speed‐induced variability: (a, b) the average LSNR and sphere count curves from 6 cm, 4 cm, and 2 cm coverage; (c, d) the box plot of the LSNR and sphere counts at the depth of 2.2 cm for all the transducer coverage ranges.

To analyze the variability induced by different transducer translation speeds, the LSNR and sphere count curves from different acquisitions under the same transducer translation speed were averaged. The average LSNR and sphere count curves from 6 cm, 4 cm, and 2 cm coverages are shown in Figures 7a and b. The LSNR and sphere count at a depth of 2.2 cm from different transducer translation speeds are shown in Figures 7c and d. Variability was calculated using the relative standard deviation across the five average LSNR/sphere count curves at different transducer translation speeds, including scanning coverages of 2 cm, 3 cm, 4 cm, 5 cm, and 6 cm over a fixed 4.13s acquisition time. The overall variabilities of LSNR and sphere counts were 5.5% and 31.3%, respectively. The variabilities at 2.2 cm were 6.1% and 31.4%, respectively. Despite the large variability of the sphere counts, the LSNR demonstrated a relatively small variability of around 5% due to different transducer translation speeds.

Noticeably, the 6 cm coverage demonstrated significantly poorer LSNR compared with the other scanning coverages in Figure 7a. This could primarily be attributed to the faster transducer translation speed, which reduced the likelihood of scanning the sphere through its maximum cross‐section, leading to diminished contrast of the spherical voids, as visually observed in Figures 5b and c. The reduced contrast, as well as the small target size, made sphere detection more difficult. As illustrated in Figure 7b, the count of spheres detected for the 4 cm coverage is approximately twice the count for the 2 cm coverage. Under ideal detection conditions, the 6 cm coverage should yield a sphere count 1.5 times that of the 4 cm coverage. However, the number of spheres detected for 6 cm remains similar to that for 4 cm. This is because although the total number of spheres within the scan increases as the coverage increases, which leads to a higher sphere count, the percentage of detected spheres decreases due to the reduced contrast. Nevertheless, the sphere counts still exhibited a nearly linear relationship with the transducer coverages with *R* ^2 ^= 0.953.

#### 3.2.3 Inter‐operator variability

**FIGURE 8:**
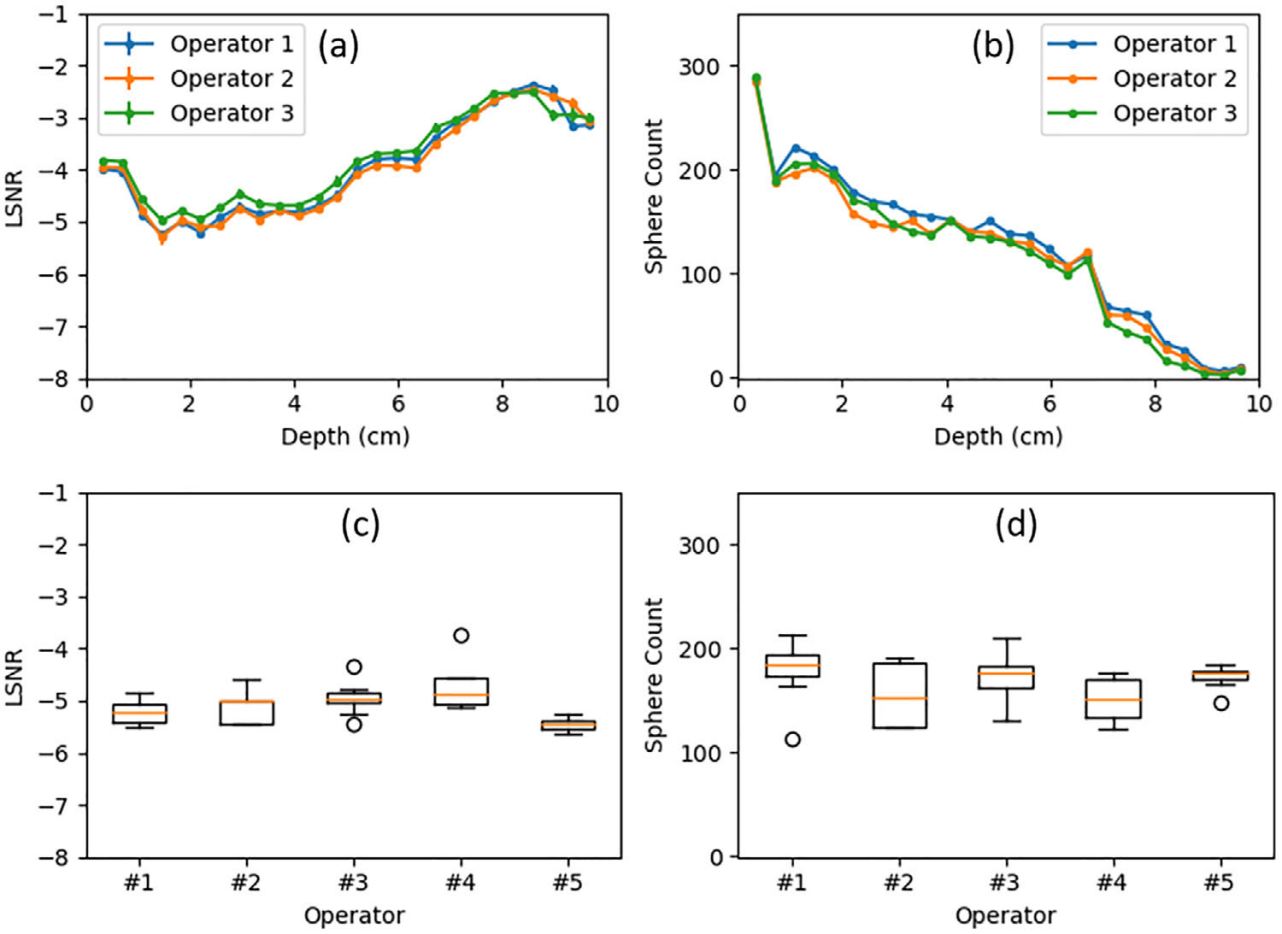
Inter‐operator variability: (a, b) the average LSNR and sphere count curves from Operators #1, #2, and #3 with 6 cm coverage; (c, d) the box plot of the LSNR and sphere counts at the depth of 2.2 cm from all 5 operators with 6 cm coverage.

A similar analysis was performed to assess inter‐operator variability. All five operators were instructed to cover 6 cm over the 4.13s acquisition time, and the inter‐operator variability was calculated based on the average of the 10 acquisitions from each operator. As shown in Figure 8, the variability of sphere counts was much smaller compared to the transducer translation speed‐induced variability shown in Figure 7. The LSNR curves exhibited very small variabilities among Operators #1, #2, and #3. Quantitatively, the overall variability of the LSNR and sphere counts were 5.2% and 8.5%, respectively. The variabilities at 2.2 cm were between 5.0% and 6.2%. The LSNRs demonstrated low variability of approximately 5% across different operators.

It should be noted that the inter‐operator variability (Figure 8) is also smaller than the intra‐operator variability (Figure 5). This is because the inter‐operator variability was calculated based on the average from 10 repeated scans of each operator, whereas the intra‐operator variability was based on a single scan.

Noticeably in Figures 8c and d, Operator #5 demonstrated a slightly better LSNR with smaller variability compared to the others. This was likely because this operator is more experienced, leading to better consistency between repeated scans. Despite differences in operator experience, the LSNR variability across all five operators remained around 5%. Furthermore, the sphere count variability due to operators was also small, likely attributable to the consistency in scanning volumes, as specified by the markers indicating the starting and ending positions on the phantom.

## 4 DISCUSSION

A RHSP and image analysis software make up an emerging, low‐cost system for testing important performance features of ultrasound systems. The proposed method enables easy freehand scanning, leading to convenient acquisition and reducing scanning time from approximately 5 minutes in mechanical scanning mode to several seconds.^12^ The convenience of freehand scanning and automated analysis grants the proposed method great potential for broad usage in routine ultrasound QA/QC.

Direct verification of LSNR accuracy would be highly labor‐intensive, as it requires manual segmentation of all spherical voids and calculation of the LSNR between the spherical voids and background accordingly. Instead, we validated the LSNR calculations by comparing them to visual impressions, as well as theoretical expectations when changing acquisition parameters. The LSNR curves complied with visual impressions in all cases. In each case, the peak of the LSNR consistently matched the depth where the spherical voids were the most distinct from the background. When comparing different settings, clearer visualization in lesion detectability consistently demonstrated a better overall LSNR curve. Changes in LSNR across different acquisition parameters also complied with theoretical expectations: higher frequency would lead to better LSNR; compounding and harmonic would lead to better LSNR; higher transmit power gave better LSNR; gain did not affect LSNR as it amplified the signals from spherical voids and background at the same time; lower dynamic range boosted LSNR due to noise saturation; and changing the transmit focal depth would shift the LSNR peak accordingly.

Our experiments would also aid in selecting scanning parameters to optimize imaging protocols in clinical practice. According to the results of the experiments, the following settings are suggested for general cases: higher nominal frequency, harmonic imaging with compounding, higher power, and a medium dynamic range. Low dynamic range is not suggested as it can lead to saturation. The gain does not influence LSNR. For the GE Logiq E9 system under dual‐focus setting, we suggest using the 2 and 3 cm dual‐focus setting as the default.

Stability is one of the major concerns for freehand acquisition. We identified three main sources of variability and analyzed each factor independently. The variability in LSNR due to intra‐operator differences, inter‐operator differences, and transducer translation speed differences was approximately 5% for each factor, demonstrating the robustness of the proposed paradigm of freehand scanning with automated image analysis software. In practice, variability due to transducer translation speed can be minimized by standardizing the scanning protocol, such as fixing the acquisition time and marking the start and end points. Hence, the combined uncertainty from intra‐ and inter‐operator factors is approximately 7%, which satisfies the needs for potential use in routine QA/QC. We did not find a significant difference between the LSNR measured by medical physicists (Operators #1 and #2) and experienced sonographers (Operators #3, #4, and #5), indicating that the QA/QC method can be easily trained and performed by ultrasound users.

In this work, a RHSP with 2 mm diameter spheres was scanned, which covered the performance assessment from the 7 to 18 MHz range.^13^ To the best of our knowledge, the proposed method is compatible with different sphere sizes and should be applicable to larger spheres, such as 4–6 mm diameters, to cover the frequency range from 1 to 7 MHz.^13^

Besides the LSNR vs. depth curve as proposed in this work, it may be extended to evaluate the elemental dropout in transducers quantitatively. Ultrasound transducers often suffer from defects that lead to slightly deteriorated images in sub‐regions. There is no consistent standard in quality control of these defects, and the removal of defective transducers is in the sole jurisdiction of sonographers and physicians. Because the units that require a quantitative evaluation would not suffer from significant dropout (otherwise, it would be obvious, and the transducer with severe element dropout should be replaced), this entire segmentation pipeline still works for dropout evaluation. It would be feasible to analyze dropout by analyzing the LSNR in the lateral direction instead of the depth direction, following a similar analysis pipeline.^19^ Establishing quantitative criteria in this way would be valuable for determining whether a transducer should be removed from clinical service or subjected to corrective actions.

## 5 CONCLUSIONS

The proposed analysis method using the RHSP eliminates the need for a mechanical scanning system and employs freehand scanning. It provides an accurate and stable quantitative assessment of lesion detectability for ultrasound performance evaluation. The accuracy of the method was validated by evaluating the performance of ultrasound systems under different imaging parameter settings and different dual‐focus settings. The method demonstrated robustness by achieving approximately 5% variability in LSNR across various transducer translation speeds and operators. With the ease of freehand, this method simplifies the image acquisition process and reduces the scanning time. It has great potential to play a critical role in routine ultrasound QA/QC in the clinical environment. It can also be used for performance evaluation and preset optimization for ultrasound scanners and probes. Future work will include extending this method to applications with curved array transducers and more complicated performance assessment tasks, such as element dropouts in array transducers.

## CONFLICT OF INTEREST STATEMENT

The authors declare no relevant conflicts of interest, aside from the disclosed employment of the author Cristel Baiu at UTune LLC, the provider of the phantom.

## Data Availability

The software implementation of the proposed RHSP‐based analytical method for evaluating LSNR vs. depth curve in ultrasound imaging is available at: https://github.com/baihui‐yu/rhsp_lsnr
